# Machine learning models for diagnosis and prognosis of Parkinson's disease using brain imaging: general overview, main challenges, and future directions

**DOI:** 10.3389/fnagi.2023.1216163

**Published:** 2023-07-19

**Authors:** Beatriz Garcia Santa Cruz, Andreas Husch, Frank Hertel

**Affiliations:** ^1^National Department of Neurosurgery, Centre Hospitalier de Luxembourg, Luxembourg, Luxembourg; ^2^Imaging AI Group, Luxembourg Centre for Systems Biomedicine, University of Luxembourg, Esch-sur-Alzette, Luxembourg

**Keywords:** Parkinson's disease, translational ML, neuroimaging, machine learning, deep learning, computer-aided diagnosis, digital health

## Abstract

Parkinson's disease (PD) is a progressive and complex neurodegenerative disorder associated with age that affects motor and cognitive functions. As there is currently no cure, early diagnosis and accurate prognosis are essential to increase the effectiveness of treatment and control its symptoms. Medical imaging, specifically magnetic resonance imaging (MRI), has emerged as a valuable tool for developing support systems to assist in diagnosis and prognosis. The current literature aims to improve understanding of the disease's structural and functional manifestations in the brain. By applying artificial intelligence to neuroimaging, such as deep learning (DL) and other machine learning (ML) techniques, previously unknown relationships and patterns can be revealed in this high-dimensional data. However, several issues must be addressed before these solutions can be safely integrated into clinical practice. This review provides a comprehensive overview of recent ML techniques analyzed for the automatic diagnosis and prognosis of PD in brain MRI. The main challenges in applying ML to medical diagnosis and its implications for PD are also addressed, including current limitations for safe translation into hospitals. These challenges are analyzed at three levels: disease-specific, task-specific, and technology-specific. Finally, potential future directions for each challenge and future perspectives are discussed.

## Introduction

Computer-aided diagnosis (CAD) systems based on medical imaging has the potential to assist clinical practice in the diagnosis of Parkinson's disease (PD). However, the suitability of CAD systems for this application is still being evaluated, and several key aspects must be taken into consideration.

The primary objective of CAD systems is not to replace radiologists and clinicians, but to support them in improving the quality and efficiency of their diagnoses (Chen et al., 2013). Although CAD systems have been in use for several decades, with successful applications in detecting pulmonary nodules (Xu et al., 1997) and breast cancer (Mangasarian et al., 1995), they were previously reliant on manual feature extraction based on domain knowledge. However, with the recent emergence of Machine Learning (ML) techniques, such as Deep Learning (DL), the automatic extraction of features from imaging data has become possible (Doi, 2007). Furthermore, the availability of large datasets and more powerful computational infrastructure has facilitated the development of advanced ML algorithms, which have the potential to significantly improve the accuracy of CAD systems (Neri et al., 2019).

Although CAD systems based on Artificial Intelligence (AI) have the potential to greatly enhance the effectiveness of clinical diagnosis and prognosis workflows, it is essential to carefully consider several key factors to ensure their safe and effective implementation in clinical practice. In fact, there is often a gap between the research literature on ML models and their final deployment in clinical applications. Closing this gap requires careful consideration and addressing several crucial aspects such as model robustness, data quality and bias, regulatory compliance, integration with existing clinical workflows, and ongoing validation in real-world settings.

A good example of a clinical deployment of an AI system that exemplified this gap is the AI-based tool by Google, Automated Retinal Disease Assessment (ARDA) system. Although this DL system was successfully developed and internally validated at the research level in 2016 (Gulshan et al., 2016), it faced several challenges in transitioning from the theoretical expectations to the reality of deploying the AI model tool in India and Thailand, as discussed in a recent paper highlighting the necessity of considering this gap (Widner et al., 2023).

While previous review papers have thoroughly covered the topic of using ML as a proof-of-concept for CAD systems (Sakai and Yamada, 2019; Mei et al., 2021), there has not been a previous review that specifically addresses the changes and potential solutions associated with the translation of these models into clinical practice for PD imaging using ML.

This review is organized as follows: first, a comprehensive background on PD, including related conditions and proposed clinical subtypes is presented. Second, the diagnosis and prognosis of PD is introduced, with a specific focus on the employment of magnetic resonance imaging (MRI). Lastly, a comprehensive analysis of the present status of computer-aided diagnosis, will be discussed, emphasizing the main limitations and future directions at three different levels. These considerations will take into account the unique features of PD, as well as the limitations of clinical brain imaging datasets, and the challenges associated with ML and DL approaches. By considering these factors, this review aims to provide insights into the potential of CAD in assisting clinical practice in the diagnosis of PD, while also highlighting the challenges that need to be addressed to ensure its safe and effective translation into clinical practice.

## Parkinson's disease and related disorders

It has been more than 200 years since the first description of the symptoms of PD by James Parkinson in his essay “The Shaking Palsy” (Parkinson, 2002). This first description refers to some of the most prominent physical landmarks of the disease, such as tremors and flexed posture. Nowadays, we have a more holistic understanding of this complex neurodegenerative disease, but currently, there is no cure, and no established biomarker for differential diagnosis of the disease (Tolosa et al., 2021).

PD is the second most common neurodegenerative disorder after Alzheimer's disease (AD), with more than 10 million people affected worldwide (Marras et al., 2018). One of the main risk factor associated with PD is advanced age. Considering that the elderly population is expected to double by 2050, the number of PD patients is expected to increase accordingly (Nerius et al., 2017). It is characterized by visible motor symptoms such as slowness of movement, muscle rigidity, and tremors at rest (Sveinbjornsdottir, 2016). However, non-motor symptoms such as depression, anxiety, cognitive deficits, sleep disturbance, hyposmia, cardiovascular problems, and bladder dysfunction can also be debilitating and may present before the motor problems (Chaudhuri et al., 2006). Notably, there is growing evidence that PD is associated with gastrointestinal dysfunction and changes to the microbiome, which may have potential as a biomarker (Elfil et al., 2020). By the time the main physical symptoms of PD appear and the patient receives a diagnosis, 30%–50% of the dopamine neurons vulnerable to PD are already lost. Hence, a key goal is to detect and quantify PD biology before their symptoms appear, during the prodromal phase (Pellicano et al., 2007). Clinical markers of this phase are non-motor and motor symptoms. Non-motor symptoms include hyposmia, constipation, REM sleep behavior disorder (RBD), excessive daytime somnolence, depression and/or anxiety, global cognitive deficit, and orthostatic hypotension. Motor symptoms include voice and face akinesia (Hustad and Aasly, 2020).

PD affects various regions of the nervous system and different types of neurons. However, much attention has been given to neurons in brain regions associated with motor symptoms, particularly the *substantia nigra pars compacta* in the midbrain. This region is involved in a critical brain pathway that facilitates movements, known as the nigrostriatal pathway (Eriksen et al., 2009). One of the most widely accepted frameworks to describe the spread of sporadic PD is Braak's hypothesis, which suggests that PD progresses through six different stages, gradually evolving from the lower brain stem to the neocortex (Rietdijk et al., 2017). The gradual degeneration of dopaminergic neurons in the substantia nigra leads to the malfunction of this pathway and the characteristic motor problems. It has been proposed that not all patients follow this progression, and two subtypes have been suggested for the disease evolution: peripheral nervous system first (PNS-first) and central nervous system first (CNS-first) (Borghammer and Van Den Berge, 2019). The existence of these subtypes is supported by *in vivo* imaging studies of RBD-positive and RBD-negative patient groups (Borghammer and Van Den Berge, 2019), as well as for genetic makers (Blauwendraat et al., 2020).

Current treatments for deficits in dopamine often involve the use of drugs that either replace or mimic dopamine in the brain (Cools, 2006). However, over time, the effectiveness of these drugs tends to diminish. In addition to medication, physical therapy can be employed as a complementary approach to enhance cognitive function in individuals with dopamine deficits (da Silva et al., 2018). Physical therapy focuses on improving mobility, balance, and coordination, which can positively impact cognitive abilities. Furthermore, alternative therapeutic avenues are being explored. Probiotics have shown potential in reducing constipation associated with Parkinson's disease (Tan et al., 2021). Additionally, anaerobic exercise has been investigated as a current approach for managing dopamine deficits (Schootemeijer et al., 2020). Moreover, emerging treatment options include drug repurposing, regenerative therapies, gene therapies, and cell-based treatments (Stoker and Barker, 2020). These innovative approaches offer promising prospects in the management of dopamine-related deficits.

Deep brain stimulation (DBS) is an effective treatment option for PD by targeting the subthalamic nucleus, globus pallidus (Lee et al., 2019), ventral intermedius nucleus (Fasano et al., 2012), and pedunculopontine nucleus (Thevathasan et al., 2018). Next-generation noninvasive DBS technologies, such as noninvasive or minimally invasive DBS (Lozano, 2017), transcranial direct current stimulation (tDCS) (Broeder et al., 2015), and transcranial magnetic stimulation (TMS) (Cantello et al., 2002), have also shown positive effects in reducing non-motor symptoms of PD when appropriate controls for side effects are in place. However, there is currently no cure for neurodegeneration, and current efforts focus on reducing symptoms to improve the quality of life.

### Related conditions

Several neurological movement disorders are closely associated with PD, and differentiating it from other diseases can be challenging, especially during the initial stages of the disease (Poewe and Wenning, 2002). Related disorders that share similar clinical features with PD can be classified into two broad categories: degenerative disorders and non-degenerative disorders (Politis, 2014). Degenerative disorders, such as Multiple System Atrophy (MSA), Progressive Supranuclear Palsy (PSP), Corticobasal Degeneration (CBD), Dementia with Lewy Bodies (DLB), and AD, can present with clinical features that overlap with PD. On the other hand, non-degenerative disorders such as Essential Tremor (ET), dystonic tremor, exaggerated physiological tremor, tremor related to hyperthyroidism, vascular parkinsonism, normal pressure hydrocephalus (NPH) (Stolze et al., 2001) and drug-induced parkinsonism can also mimic some of the clinical features of PD.

### Parkinson's disease clinical subtypes

The clinical and neuropathological heterogeneity of PD patients is well known, and consequently there have been many attempts to identify different subtypes. Initial approaches consisted of empirical classifications using *a priori* hypotheses (Zetusky et al., 1985; Jankovic et al., 1990). In recent years, research works have progressively employed data-driven cluster analysis that includes longitudinal assessment of motor and non-motor symptoms (De Pablo-Fernández et al., 2019; Zhang et al., 2019; Dadu et al., 2022). This classification method looks promising for informing patients about the future progression of the disease and for personalizing treatment. However, these criteria are not yet applied in clinics since more research is needed to unify and validate the criteria using well-curated longitudinal cohorts. Among the multiple attempts to separate the disease, several criteria have been applied, including early-onset vs. late-onset (Riboldi et al., 2022) slow vs. fast progression, with or without dementia or tremor-dominant vs. gait-dominant (Dadu et al., 2022).

### Machine learning, deep learning and computer vision

**Figure 1:**
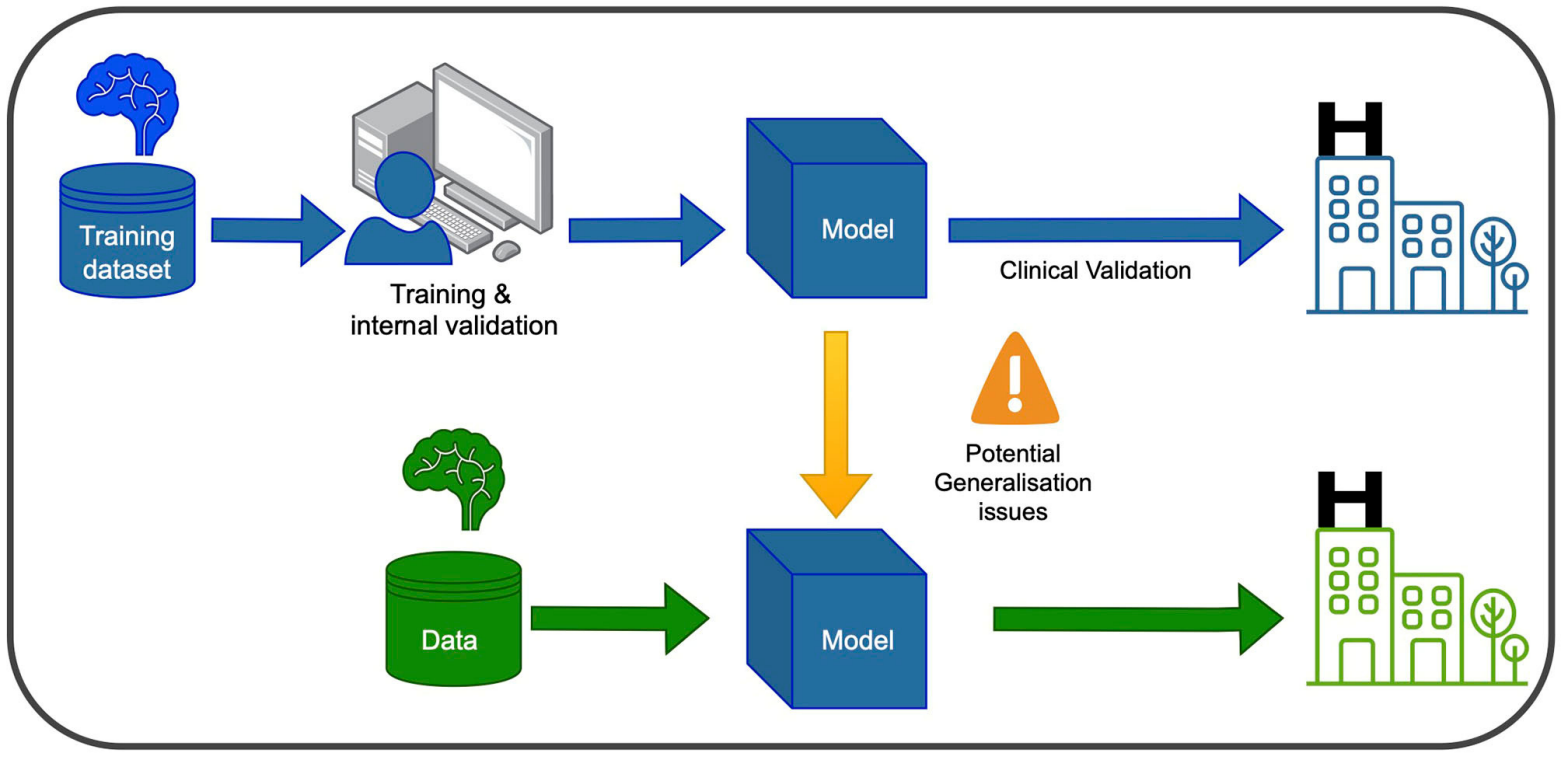
Training and using an ML model in the clinic involves two main phases. In the blue phase, the model is trained and validated using data from the same hospital. This ensures it learns from the hospital's specific context and performs well within that setting. After this, the model undergoes clinical validation to ensure its reliability and safety before deployment. In the green phase, the model can be used in new hospitals, but caution is needed to address potential generalization issues. Variations in healthcare systems and patient populations may affect its performance. Thorough testing and evaluation are necessary to ensure accurate and safe application in different healthcare settings.

In recent years, ML and DL have gained significant attention in healthcare and medical research. These computational tools enable the analysis of large and complex datasets to learn patterns and relationships, with DL algorithms utilizing multiple layers of artificial neural networks to extract abstract data representations such as images. Furthermore, Computer Vision (CV) seeks to enable computers to interpret and understand visual information from the surrounding environment. Supervised learning is a common type of ML employed in PD research, where labeled datasets are used to train the algorithm to make predictions on unseen data. Convolutional neural networks (CNNs) are the most frequently used type of neural network for image recognition to conduct tasks such as classification in medical imaging. In Figure 1, a graphical representation of the training and development of a ml-based system for clinical use is depicted.

The quality of data and labels are crucial factors that can significantly impact the performance of ML models. In current ML models, data is the most important component as the models learn from the data presented to them. Therefore, the quality of the data used in the training process is crucial. Other factors that can influence the quality of models include the choice of ML algorithms, feature engineering, hyperparameter tuning, and model selection. In addition to data quality, the quality of labels is also critical. Poor quality labels can result in biased models, incorrect predictions, and suboptimal performance. Moreover, data representation is equally important for a good model performance. A training set should be a representation of the event that we want to model, and a good validation strategy is essential for assessing the generability of the model.

## Parkinson's disease diagnosis and prognosis

**Figure 2:**
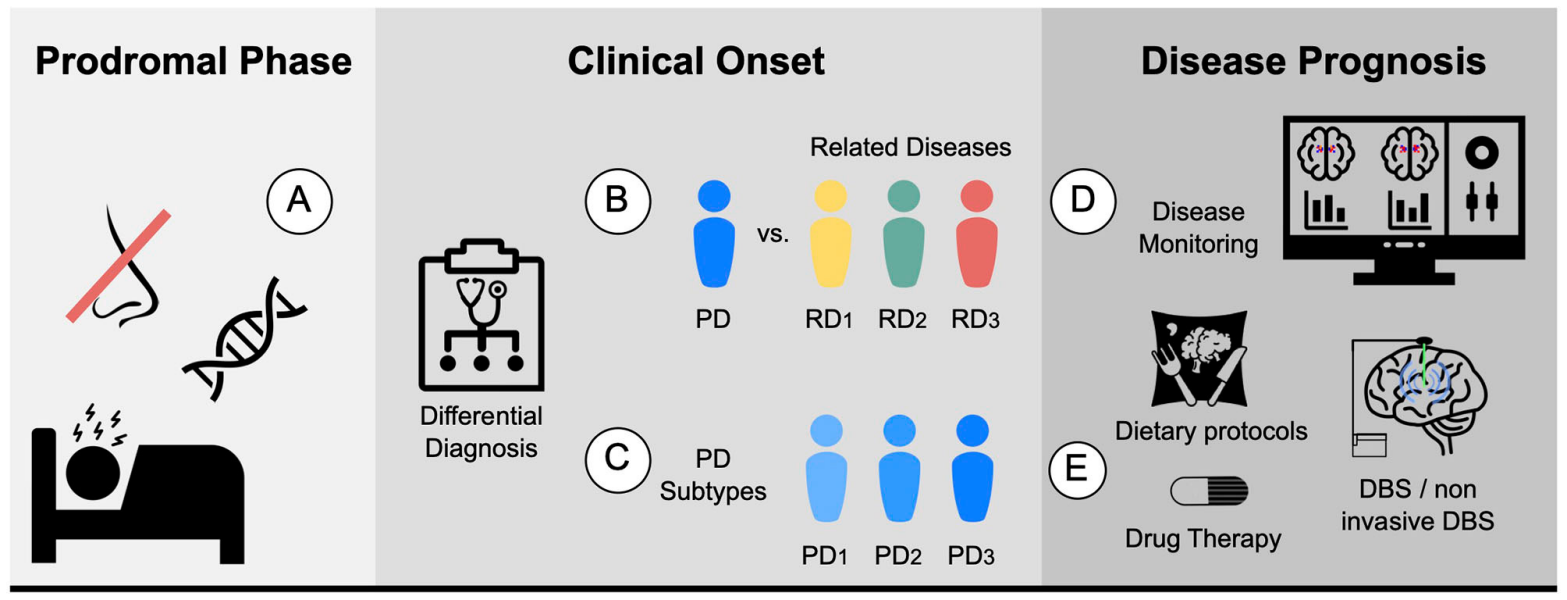
Proposed biomarkers for PD using MRI: **(A)** Prodromal biomarker: identifying brain changes during the prodromal phase. **(B)** Differential diagnosis biomarker: assisting in distinguishing PD from related diseases. For instance, ET or MSA. **(C)** Subtype biomarker: classifying PD patients into their corresponding subtypes. **(D)** Progression biomarker: aiding in predicting the progression of the disease and treatment response with disease monitoring. **(E)** Therapy response biomarker: facilitating personalized medicine by finding the best drug, dietary protocols, physical or cognitive therapies, and predicting the potential response to other therapies such as DBS and non-invasive DBS.

Accurate diagnosis of PD is essential, and achieving enough specificity to distinguish between similar conditions during the clinical phase is crucial. Developing monitoring tools to track disease progression and evaluate individual patient response, including the presence and magnitude of treatment side effects, is also necessary. Furthermore, quantifying the different systems, such as motor, memory, and limbic system, could help stratify patients. In terms of prognosis, ongoing efforts are focused on establishing clear criteria for patient stratification into different subtypes, which would aid in the development of targeted treatment approaches. Figure 2 proposes five different biomarkers that are relevant in the context of PD.

The current diagnostic criteria for PD is biased on a comprehensive evaluation of a patient's clinical presentation and medical history. Given the lack of a definitive diagnostic test for PD, clinicians rely on a variety of subjective and objective measures to make an accurate diagnosis. Clinical evaluation, involving detailed inquiry into the patient's symptoms, medical history, and family history, represents a fundamental component of the diagnostic process. Alongside this, a thorough physical examination aimed at assessing motor function, including muscle strength, reflexes, and coordination, as well as cognitive function and mood, is also typically conducted. To support a clinical diagnosis, objective tests may be employed. Imaging modalities such as MRI or computed tomography (CT) scans are typically employed to rule out other conditions that may present similarly to PD. Furthermore, nuclear imaging techniques such as Single Photon Emission Computed Tomography (SPECT) and Positron Emission Tomography (PET) can serve to buttress the diagnosis of PD.

Nowadays, there is a significant effort to find biomarkers for PD. In the preclinical phase, it highlights biomedical markers, such as those that measure the activity of mitochondria dysfunction and oxidative stress (He et al., 2018). Others focus on measuring abnormal protein aggregation and accumulation, such as alpha-synuclein (Foulds et al., 2011) or tau protein (Constantinescu and Mondello, 2013). Some try to measure established clinical features such as olfactory dysfunction, RBD, or constipation. During the prodromal phase, genetic biomarkers have been explored, such as mutations in Parkin (Pickrell and Youle, 2015), Leucine-rich repeat kinase 2 (LRRK2) (Tolosa et al., 2020), or Alpha-synuclein (SNCA) (Mata et al., 2010). Finally, neuroimaging techniques are also promising.

In the context of brain imaging, a biomarker is an objective characteristic derived from an *in vivo* image that measures a normal biological process, pathological process, or response to a therapeutic intervention (Mohammadi, 2013). It must fulfill the following criteria: be quantitative, repeatable, reproducible, precise, reliable, sensitive, and specific, and be measured on a ratio or interval scale (Smith et al., 2003).

### Medical imaging in Parkinson's disease

The main advantage of brain imaging is that it allows for the visualization of the functional and structural brain changes that result from underlying pathophysiological abnormalities (Saeed et al., 2017). There are several imaging techniques that can be used to aid in the diagnosis and prognosis of PD.

On the one hand, there is a set of non-invasive techniques for investigating PD, such as **structural magnetic resonance imaging** (MRI) with T1, T2, and susceptibility-weighted sequences, which allow for volumetric and voxel-based morphometric analyses, as well as MRI-derived visual signatures (Saeed et al., 2017; Chougar et al., 2021). For instance, Schwarz et al. (2014) proposed that the appearance of the dorsolateral substantia nigra as a “swallow tail” shape on high-resolution, iron-sensitive, MRI at 3T, where healthy nigrosome-1 appears as a characteristic feature that could be employed as a marker of degeneration in that area. Further, a promising structural MRI sequence for PD diagnosis is neuromelanin-sensitive MRI (NM MRI), which can detect neuromelanin, a pigment synthesized by the substantia nigra dopamine neurons that is lost when neurons die in PD patients. NM's avid binding of iron enables its detection via magnetic resonance imaging (Sulzer et al., 2018). The use of NM MRI to define regions of interest (ROIs) in the substantia nigra pars compacta (SNpc) has shown promising results compared to using T2*-weighted contrasts. This approach has yielded consistent results, and studies have found that the mean R2* in the SNpc, as defined by neuromelanin-sensitive MRI, was significantly increased in PD patients (Langley et al., 2019).

**Diffusion tensor MRI** (DT-MRI) is another technique used to study the structural connectivity of the brain in PD. DT-MRI investigates the integrity of white matter tracts connecting different brain regions, and studies have shown that it can detect changes in white matter connectivity in PD patients. Specifically, Yoshikawa et al. (2004) demonstrated that DT-MRI can detect the loss of fractional anisotropy (FA) in the nigrostriatal projection, indicating that more than half of the dopaminergic neurons in this projection may be lost before the onset of PD.

Furthermore, **functional magnetic resonance imaging** (fMRI) can detect changes in blood flow in response to neural activity, which enables researchers to study brain function. In PD, fMRI has been used to investigate changes in brain activity related to both motor and non-motor symptoms. For instance, Tahmasian et al. (2015) employed resting-state (rs-fMRI) to assess the effect of dopamine replacement therapies, such as levodopa and dopamine agonists, on PD patients. Additionally, researchers have used fMRI techniques to investigate the effect of DBS therapy in the modulation of specific brain regions. An example of this is a study by Boutet et al. (2021), in which fMRI brain response patterns were used to predict the optimal parameters for DBS by identifying patterns associated with clinically effective stimulation that preferentially engages the motor circuit.

Additionally, **Transcranial sonography** (TCS) is an ultrasound-based neuroimaging technique that utilizes low frequency sound waves to generate images of the brain. In the context of PD diagnosis, TCS has been employed to investigate the structure and function of the SN, among other brain regions. Mahlknecht et al. (2013) demonstrated that TCS exhibits favorable diagnostic accuracy in detecting PD subjects based on the presence of hyperechogenicity in the SN Furthermore, TCS has been investigated as a potential tool to establish disease progression biomarkers that could provide real-time feedback on the rate of dopaminergic neuronal death in animal models (Zhang et al., 2020).

On the other hand, invasive **molecular imaging** techniques such as PET and SPECT can detect reduced density of dopaminergic nerve terminals in the basal ganglia. PET is an *in vivo* functional neuroimaging technique that utilizes a variety of radionuclides to assess the integrity of the dopaminergic system, cerebral metabolism, pathological protein accumulation, and inflammation in the brain (Saeed et al., 2017). Radiotracers, such as 18F-dopa (Morrish et al., 1996) and 11C-raclopride (Politis et al., 2008), can image the integrity of presynaptic and postsynaptic nigrostriatal and hypothalamus projections, respectively. Using SPECT, dopamine transporter SPECT (DAT SPECT) imaging is an objective tool for assessing dopaminergic function of presynaptic terminals, differentiating parkinsonian disorders related to striatal dopaminergic deficiency from those not related. DAT SPECT imaging can confirm or exclude a diagnosis of dopamine-deficient parkinsonism and detect dopaminergic dysfunction in presymptomatic subjects at risk for PD. Normal DAT SPECT findings exclude presynaptic striatal dopaminergic insufficiency, while abnormal findings indicate a variety of diseases with this insufficiency as a common pathophysiological process (Akdemir et al., 2021). For instance, DaT SPECT imaging with (123I)ioflupane is a useful tool to distinguish between PD-tremor and non-PD tremor, such as ET (Bajaj et al., 2013). Besides, other non-dopaminergic imaging techniques such as glucose metabolism and PDE10A expression have been proposed to study PD (Pagano et al., 2016). Additionally, extrastriatal ^123^I-FP-CIT SPECT impairment has been proposed to detect early cases of PD (Nicastro et al., 2020).

While imaging techniques are currently used for research purposes and can assist in challenging cases, they are not commonly used for diagnosing PD. However, it is worth noting that most PD diagnoses do not involve imaging. In the future, brain imaging could be integrated into the diagnostic process as advancements in techniques like ML and CV hold promise for improving the analysis of imaging data. These developments may enable more accurate and reliable diagnostic applications of imaging in PD.

## Computer-aided diagnosis using brain imaging: main limitations and future directions

**Figure 3:**
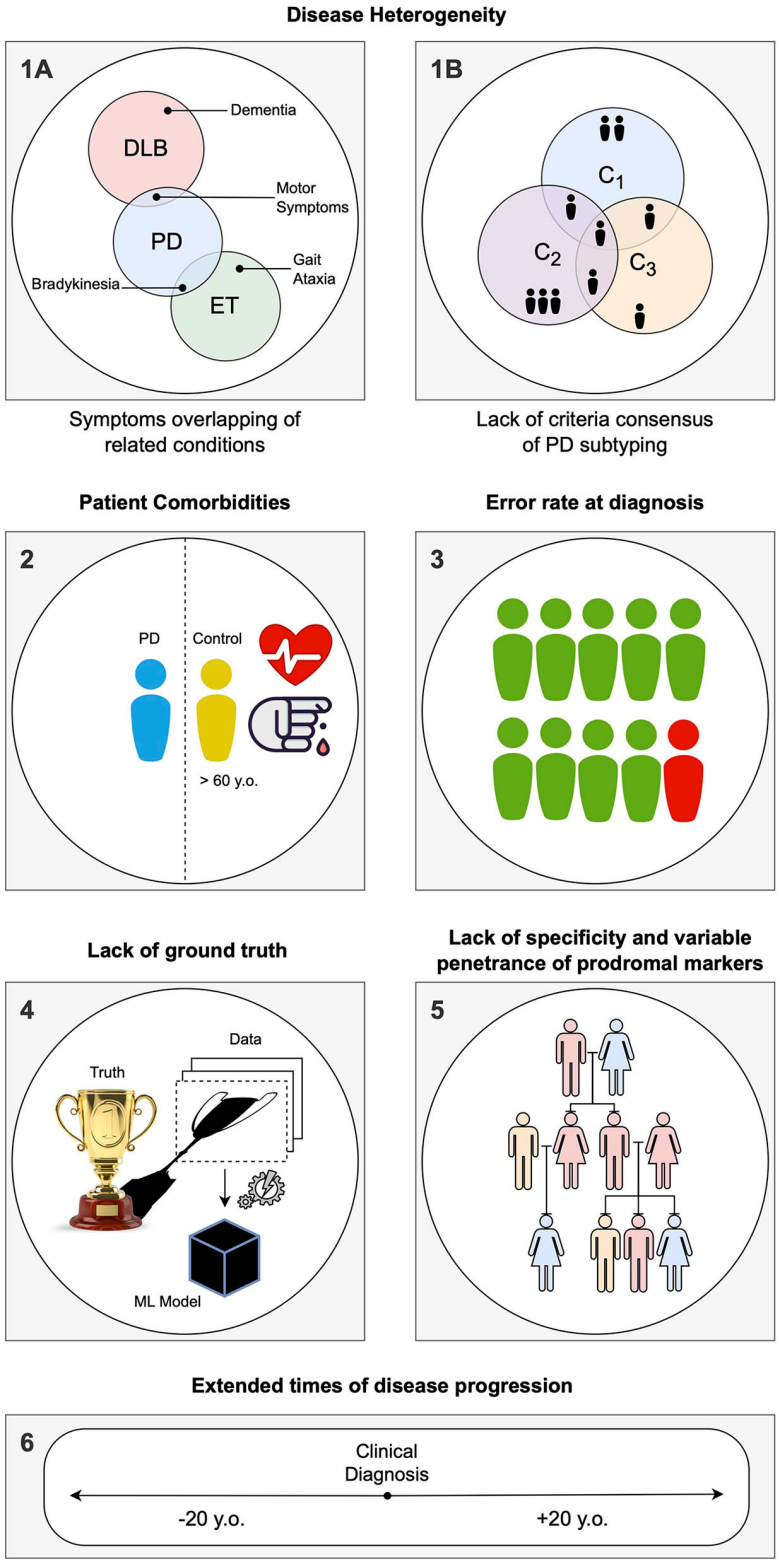
Summary of the specific limitations in computer-aided diagnosis (CAD) for Parkinson's disease (PD) associated with idiosyncrasies of the disease, as addressed in Section Limitations associated with Parkinson's disease: **(1)** During the labeling of datasets for supervised learning, several problems can be encountered. **(1A)** Building a solution for differential diagnosis can be challenging due to the overlapping symptoms of PD and related disorders. This challenge is especially significant during the initial phases of clinical diagnosis, where such solutions would be most useful. **(1B)** PD is known to have several subtypes with implications for clinical treatment, but there is a lack of clear global consensus, adding another layer of complexity. **(2)** PD being an age-related disorder, the control subjects used in age-pairing may have additional health conditions or factors that can affect their representatives as healthy individuals. **(3)** Due to the complexity of PD, there is a notable rate of misdiagnosis, even in specialized centers, particularly during the early phases of clinical diagnosis. This hampers the accuracy of labels used in supervised learning solutions. **(4)** When acquiring data and building a model, a simplification of the disease within the context of human biology is necessary, as it is the case with any other data-driven solution. Consequently, any developed solution will have errors, particularly if the model is used in different conditions than those it was designed for. **(5)** Detecting PD in the prodromal phase is particularly challenging. A common approach is to employ known markers that increase the probability of developing the disease, such as genetic mutations. However, the specificity of these markers to PD is variable. **(6)** Conducting long-term longitudinal studies that are consistent in terms of acquisition protocol while maintaining low levels of drop-out rates is extremely difficult for PD, given its nature as a complex, long-term neurodegenerative disease.

The main limitations of CAD systems in the context of PD can be grouped into three categories. The first set of limitations represented in Figure 3 pertains to the particularities of PD, its diagnosis, and prognosis. The second set of limitations is associated with the characteristics of datasets consisting of brain imaging. These limitations include factors such as the heterogeneity of the imaging modalities used, variability in image acquisition protocols, challenges in image preprocessing and feature extraction, and issues related to sample size and data quality. The third set of limitations is associated with the use of ML/DL-based algorithms for CAD systems. These limitations include challenges such as overfitting, lack of interpretability, bias and generalization issues, and difficulties in integrating multiple data sources. A summary of the main limitations can be found in Table 1, which will serve as a reference point throughout the discussion of potential solutions to address these limitations.

**Table 1:** Overview of limitations and future directions at the three levels: disease-specific, task-specific, and technology-specific.

**Limitations**	**Directions**
**Parkinson's disease**	
Disease heterogeneity	Considering subgroups of PD and careful assessment of controls
Patients' comorbidities	Large and Long studies and control of unwanted correlations
Error rate at diagnosis	Acknowledging errors and employing noise-labeled techniques
Extended times of disease progression	Institutional incentives, importance of consistency in protocols
High variability of prodromal markers	Multimodal prodromal markers, epigenetics changes
Lack of ground truth	Objective measures, holistic multidisciplinary approach
**Clinical brain imaging datasets**	
Complexity of brain imaging	Multimodal approach, combination with clinical measures
Lack of standardization in acquisition	Standardization of acquisition, sharing study assumptions
Lack of standardization in preprocessing	Sharing raw data and reproducible code ability
Lack of standardization in annotation	Assisted annotation with guidelines and unsupervised learning
**Machine learning/deep learning**	
Generalization issues	Avoid overfitting, control for spurious correlations
Algorithmic Bias	Acknowledge algorithm bias and prioritize fairness strategies
Need for better interpretability	Prioritize transparency and ethics, GDPR compliance
Model explainability	Use explainable ML algorithms, employ interpretability methods
Model uncertainty	Documentation of uncertainty sources, calibration methods
Costly systems to develop and maintain	Pre-train models, cloud computing, decentralized ML
Security and privacy challenges	Proactive security and privacy strategies

CAD systems have the potential to improve the accuracy and efficiency of diagnosing various diseases. By analyzing medical imaging data, genetic data, and clinical data, these systems can identify patterns and biomarkers associated with the disease that may be difficult to detect otherwise, which can accelerate the diagnostic and treatment workflows in clinical pathways. Moreover, CAD systems can be employed to evaluate disease progression, measure therapeutic responses to drugs in clinical trials, and speed up the development of new treatments.

Other benefits of CAD systems include the objectification of diagnosis, as the current diagnosis relies on subjective evaluation of motor and non-motor symptoms, making CAD systems promising tools for the objective evaluation of symptoms. In the context of MRI for PD, CAD systems can provide quantitative measures of the changes associated with the disease at physical, functional, and metabolic levels. Furthermore, the employment of CAD systems could aid in the unification of clinical diagnosis criteria. Additionally, CV solutions, including those that employ DL as an optimisation technique, have been shown to excel at detecting subtle changes and complex patterns in comparison with human vision. Therefore, CAD systems have the potential to serve as a valuable second or supporting opinion, as they do not experience a reduction in productivity over time, as can happen with human experts.

There are many research-level papers proposing proof-of-concept approaches for CAD systems in PD, emphasizing the importance of robust models. For instance, Castillo-Barnes et al. (2018) utilized the PPMI dataset and proposed an Ensemble Classification model to classify PD patients. Similarly, Augimeri et al. (2016) demonstrated the potential of support vector machines in combination with careful feature extraction to analyze DaTSCAN scans for PD applications. In line with these studies, Mart́ınez-Murcia et al. (2014) also proposed a PD classification method using DaTSCAN scans.

Similarly, machine learning (ML) has been employed to distinguish between PD and related disorders. For instance, Talai et al. (2021) propose a multimedia approach using T1-weighted, T2-weighted, and diffusion tensor imaging (DTI) to aid in the differential diagnosis of progressive supranuclear palsy Richardson's syndrome (PSP-RS). In the same vein, Martins et al. (2021) reported on the use of PET uptake and MRI for distinguishing Parkinsonian syndromes. Similarly, Castillo-Barnes et al. (2020) conducted a study that employed SPECT scans from the PPMI database and compared different ML methods.

More recently, CNN has been successfully proposed for the classification of brain imaging in PD. For instance, Chakraborty et al. (2020) proposed a classification using T1 weighted MRI scans using CNNs. Similarly, Martinez-Murcia et al. (2019) demonstrated the use of autoencoders to classify complex neurological diseases such as Alzheimer's. Finally, Shinde et al. (2019) also demonstrated the potential of CNNs in the modality of neuromelanin-sensitive MRI with great performance (Biondetti et al., 2020).

The mentioned research-level papers and alike ones, provide a valuable insights into the potential of CAD systems for PD. However, it is crucial to acknowledge that these studies primarily focus on demonstrating the effectiveness of specific methodologies or models in isolated aspects of PD diagnosis or classification. While their findings are promising and essential to the progress in the area, they represent only a fraction of what is required for the development of comprehensive and practical clinical systems.

To build end-to-end clinically useful CAD systems for PD, various aspects need to be considered beyond the individual proof-of-concept models. These aspects may include data acquisition and quality assurance, integration with existing clinical workflows, interpretability of the models, regulatory compliance, ethical considerations, scalability, and validation in diverse patient populations. The following sections of the paper will delve into these critical considerations and discuss potential solutions to ensure the successful implementation and utilization of CAD systems in real-world clinical settings.

## Limitations associated with Parkinson's disease

### Disease heterogeneity: intra-class variance and inter-class similarity

Medical conditions may have several etiologies. Moreover, one etiology may lead to more than one disease (Coleman and Tsongalis, 2009). Consequently, medical conditions are commonly defined clinically or pathologically (instead of etiologically). PD presents high variability at both prodromal and clinical phases (He et al., 2018). We can refer to this variability as an intra-class variance. However, another level of complexity exists due to the overlap of PD symptoms with those from other diseases, which calls for thorough differential diagnosis (Kalia and Lang, 2015). For instance, patients with arterial hypertension may exhibit distinct neuroimaging abnormalities detectable by brain MRI (van Veluw et al., 2014), which may complicate the diagnosis of PD using medical imaging techniques in these individuals. Thus, we can find a high inter-class similarity. Finally, diseases are described based on a definable deviation from a normal phenotype made evident through symptoms, and pathological markers, to then become grouped into categories. However, studies and taxonomies struggle to find a consensus for PD subtypes (Albrecht et al., 2022). Hence, studies may employ different subtypes to refer to the same biological mechanism and therapy response.

### Patients' comorbidities

In addition to the aforementioned complexity, the onset age of PD in patients is typically around 60 years, making it difficult to differentiate symptoms caused by aging and other comorbidities from those of PD (Deeb et al., 2019). For instance, common comorbidities in PD patients, such as hypertension and diabetes, have an unknown effect on the pathogenesis and progression of PD (Santiago et al., 2017). This presents a twofold challenge: first, it complicates the identification of a reliable set of control and diseased subjects, making it difficult to distinguish between groups. Second, due to the lack of knowledge regarding the effects of comorbidities on PD onset and development, controlling for these characteristics is challenging. As a result, researchers may face a “lose–lose” situation, as ML models may make assumptions that cannot be refuted or confirmed by the researcher. This situation is also referred to as butterfly bias, in which a variable or feature may be considered both a confounder and a source of M-bias (Ding and Miratrix, 2015).

To mitigate the effects of comorbidities and the heterogeneity of PD, researchers often employ large sample sizes to account for the variability in the population and the disease. For example, datasets like the Parkinson's Progression Markers Initiative (PPMI) (Marek et al., 2011) and the Oxford Parkinson's Disease Centre discovery cohort (OPDC) (Lawton et al., 2015) acknowledge the presence of subtypes and follow patients over extended periods, presenting clinical data in addition to imaging data. Moreover, studies frequently use statistical techniques such as propensity score matching (Huang et al., 2013), stratification (Virreira Winter et al., 2021), and multivariable regression (Pechevis et al., 2005) to control for confounding variables. Another approach is to utilize ML algorithms that can handle multiple confounders and nonlinear relationships between variables, such as random forest (Oprescu et al., 2019) or support vector machine models (Westreich et al., 2010).

### Error rate at diagnosis

The aforementioned challenges are further compounded by the difficulty of accurately diagnosing PD. According to Hess and Okun (2016), the misdiagnosis rate of PD can range from 10 to 20% or greater, depending on clinician experience. Other studies have reported misdiagnosis rates of 20%–30% in the early stages, with the main causes being the failure to recognize atypical parkinsonian disorders such as dementia with Lewy bodies or multiple system atrophy (Poewe and Wenning, 2002). Consequently, researchers must address the challenges of training models with noisy labeled data (Karthik et al., 2021), where label noise can potentially degrade model performance.

To address noisy labeled data several approaches have been proposed, including semi-supervised learning, where a small set of labeled data is combined with a large set of unlabeled data to improve the model's accuracy (Adeli et al., 2018). Another approach is active learning, where the model is iterative trained on a small set of labeled data, and the most informative samples are selected for annotation by a human expert, reducing labeling costs while maintaining or even improving the model's accuracy (Settles, 2009; Garcia Santa Cruz et al., 2022a). Recent developments in DL have led to the emergence of new techniques that can handle label noise more robustly, such as the label Smoothing technique (Müller et al., 2019) that reduces the impact of noisy labels on the loss function by smoothing the label distribution. Ensemble techniques also help mitigate the impact of label noise on model performance by combining the predictions of multiple models, each trained on a slightly different subset of the data (Adeli et al., 2018).

### Extended times of disease progression

PD is characterized by a slow progression, with a period of up to 20 years before the clinical phase (Kalia and Lang, 2015), and can survive up to 20 years in the clinical phase (Hassan et al., 2015), with a mean survival onset of 12 years (Rajput, 1992). This slow progression impacts longitudinal follow-up of study participants, which becomes difficult and prone to high dropout rates and protocol changes. It also brings another important dimension into play, as data subjects may showcase both different ages and distinct PD stages. Moreover, assumed control subjects may reveal PD symptoms in the long term, increasing the risk of ascertainment bias.

The extended duration of longitudinal studies can lead to higher rates of dropout and protocol changes. To mitigate these issues, researchers can employ remote monitoring technologies that allow patients to be monitored from their homes, reducing the need for in-person visits. Wearable sensors can also provide continuous, objective measurements of symptoms and mobility (Kubota et al., 2016; Arroyo-Gallego et al., 2018). Additionally, providing incentives to patients and institutions can help improve retention rates (Smith et al., 2019). For brain imaging studies, it is important to maintain consistent imaging protocols and analysis methods to reduce the risk of acquisition bias (Castro et al., 2020).

### Lack of specificity and variable penetrance of prodromal markers

Finding markers for the prodromal phase of PD is complex in many aspects. One of the key factors hindering the discovery of such markers is the low frequency of the disease, which is estimated to be under 2% (Muangpaisan et al., 2011). This low frequency makes it challenging to find participants in the prodromal phase of the disease, as large sample sizes are required for such studies. To overcome this challenge, researchers often employ non-specific markers to identify individuals who may be in the prodromal phase of PD. These non-specific markers include rapid eye movement sleep behavior disorder (RBD), hyposmia (reduced ability to smell), depression, gastrointestinal symptoms, and mild motor symptoms. However, the use of non-specific markers has limitations, as they are not specific to PD and may be present in individuals who do not develop the disease (Durcan et al., 2019). Although specific markers such as genetic markers have been identified, their use is limited by their variable penetrance, which is often incomplete and dependent on the population. Some of the most commonly associated genes with PD are LRRK2, Glucocerebrosidase (GBA), and SNCA (Niotis et al., 2022). This means that even if an individual has a genetic marker associated with an increased risk of developing PD, there is still a significant chance that they may never develop the disease.

Finding markers for the prodromal phase of PD is complex, but one potential solution to overcome the challenge of low disease frequency and the need for large sample sizes is to collaborate with multiple research centers and establish consortium. Another approach to identifying specific markers for the prodromal phase of PD is to consider multiple sources of data, such as the hyposmia test (Siderowf et al., 2012). Finally, to address the limitations of genetic markers with incomplete penetrance, researchers can focus on identifying epigenetic modifications associated with the prodromal phase of PD, which may provide more accurate and specific markers for early detection of the disease (Chen and Ritz, 2018).

### Lack of ground truth

In addition to the challenges of finding markers for the prodromal phase, there are also challenges related to generating accurate ground truth data for supervised learning. PD is not fully understood yet, which can lead to errors in the models. Deliberate idealisations are inherent in any model, but inaccurate assumptions based on insufficient knowledge can lead to biased and inaccurate representations. An example of this is the lack of understanding about comorbidity effects. Disparities in these regards can affect coherence between studies, as causal assumptions may vary across research teams and over time. Conducting further research on the disease could be a potential solution to enhance the understanding of the disease. This research can include a better understanding of the various aspects that contribute to the disease, such as adopting a complex systems approach (Cohen et al., 2022). Another solution is to develop more objective and quantitative measures of motor symptoms using wearable sensors and digital technologies.

Current diagnosis relies on assessments by physicians, often employing the current gold standard, the Unified Parkinson's Disease Rating Scale (UPDRS) (Movement Disorder Society Task Force on Rating Scales for Parkinson's Disease, 2003). Furthermore efforts are underway to develop more objective and continuous measures of motor symptoms using wearable sensors and digital technologies (Parisi et al., 2015; Lu et al., 2021). These emerging technologies can provide more accurate and reliable data for the diagnosis and monitoring of PD (Kubota et al., 2016). By replacing subjective evaluations with objective measurements, the accuracy of diagnoses may be improved, leading to earlier identification and treatment of PD. Further research on the missing link between genetic and environmental causes of the disease can also contribute to a better understanding of PD (Hill-Burns et al., 2017). Additionally, standardizing diagnostic criteria and protocols across research teams and clinical settings can increase coherence between studies and improve the accuracy of the diagnosis. One such criterion is the UK Brain Bank criteria (Postuma et al., 2018). Enhanced collaboration and communication between researchers and clinicians may serve as a valuable means to reinforce the aforementioned efforts.

## Limitations associated with clinical brain imaging datasets

### Diversity and complexity of *in vivo* imaging brain markers

The pathology underlying PD motor symptoms such as tremors and bradykinesia is mainly associated with the loss of dopaminergic neurons in the *substantia nigra* and other gray matter alterations visible through brain imaging. However, non-motor symptoms of PD such as hyposmia, sleep disturbances, and depression do not present a clear *in vivo* imaging brain marker, even though some NMS-related brain alterations have been described. In particular, Prell (2018) state that imaging NMS characteristics may require different modalities, e.g., rs-fMRI for fatigue, fMRI and FDG-PET for mild cognitive impairment. In addition, studies have shown that quantitative iron imaging techniques such as R2*, SWI, and QSM are reliable markers of iron content in PD. These measurements have also been found to correlate with the severity of motor symptoms. Among these techniques, QSM has been identified as more robust and reproducible than R2* and is more adequate for use in multicenter studies (Pyatigorskaya et al., 2020). Finally, some authors have even discouraged the routine use of neuroimaging techniques in clinical practice for PD (Pagano et al., 2016). As stated by Pagano et al. (2016), “despite significant evidence for the utility of neuroimaging in assessing parkinsonian patients, none of the neuroimaging techniques is specifically recommended for routine use in clinical practice.”

Therefore, the impact of this variety is threefold. First, the symptoms may not associate with structural or functional brain patterns. Second, when existing, such patterns require particular brain imaging modalities. Finally, such patterns may not be specific to PD. On top of these three circumstances, the temporal evolution of the disease adds another layer of complexity. Each stage calls for different symptoms, which in turn require dedicated imaging modalities with different diagnosis specificity. In this light, accurate PD subtyping becomes challenging, as obtaining a complete view of the brain manifestations of PD symptoms requires image acquisition of several modalities or the employment of multimodal approaches (Saeed et al., 2017; Chougar et al., 2020; Albrecht et al., 2022).

One potential solution to address this issue is to use a combination of multiple imaging techniques. Multimodal approaches can provide a more complete and accurate picture of the disease by capturing different aspects of brain function and structure, as well as the density of neurotransmitter receptors such as dopamine receptors. Additionally, clinical assessments can be supplemented by specific neuropsychological questionnaires or physiological tests, with subsequent confirmation by imaging or a biochemical marker, as different modalities are suitable at different stages of disease progression (Michell et al., 2004). Moreover, the use of multi-modal data, combining clinical, motor, cognitive, and neuroimaging data, can aid in subtyping PD and potentially identifying correlations between the pathology manifested in the brain and the motor and non-motor symptoms of the patient (Albrecht et al., 2022). However, it is important to note that using multiple imaging modalities can also pose some challenges, such as the need for specialized expertise, the complexity of data integration (Behrad and Abadeh, 2022), and the increased cost and time required for imaging and analysis.

### Lack of standardization in acquisition, preprocessing, and annotation pipelines

After image acquisition, another set of problems may compromise research. First, variations in the acquisition parameters may alter the observed changes in longitudinal studies. Chua et al. (2015) showed how variability in MRI acquisition parameters between scans can confound observations. Then, the diversity of preprocessing pipelines across studies presents another dimension for potential unwanted interactions and errors. For instance, the exclusion criteria for head motion may vary across studies without common criteria. Strother (2006) highlighted how the preprocessing steps interact with every decision taken during the design and execution of fMRI experiments. The authors argue that “applying a new processing pipeline to a raw dataset may result in significantly modified spatial activation patterns as a result of changing/optimizing preprocessing techniques and/or the data analysis approach.” Similarly, Power et al. (2017) identified several contributors to global fMRI signals such as hardware artifacts and head motion that were not removed from scans through denoising techniques, affecting the observed covariances. Bhagwat et al. (2021) underscored the variability introduced by preprocessing in neuroimaging pipelines. Hence, the lack of standardization in acquisition, preprocessing, and annotation pipelines can lead to unwanted interactions and errors, which has significant implications for the reliability and reproducibility of neuroimaging research (Brauneck et al., 2023).

To address this issue, it is crucial to develop and validate standardized protocols and criteria for data acquisition, preprocessing, and analysis. This can be achieved through a variety of approaches, such as establishing international consortia, promoting open data sharing, and providing training and resources for researchers. For example, the International Society for Magnetic Resonance in Medicine (ISMRM) has developed several standards for MRI data acquisition and analysis, including quantitative MR (Weingärtner et al., 2022). In addition, promoting open data sharing and encouraging researchers to openly share their raw data and analysis pipelines can help to identify potential sources of variability and errors in data processing and analysis. This can facilitate the development of more robust and reliable methods for data preprocessing and analysis. Several initiatives have already been developed to promote open data sharing in neuroimaging, such as the OpenfMRI (Poldrack et al., 2013) and NeuroVault (Gorgolewski et al., 2015) repositories. Furthermore, educating researchers about the importance of standardization in neuroimaging research (Laird et al., 2011) and providing them with the necessary tools and resources to implement standardized protocols and criteria in their research is crucial, including standardization of the metadata as a way to reflect the causal and anti-causal assumptions made during the data collection and annotation (Garcia Santa Cruz et al., 2022b). Further, standardization of the annotation pipeline is important to improve the consistency and quality of annotations. To tackle this issue, it is important to have standardized guidelines and procedures. This can reduce misinterpretation, which may result in inconsistency, making the subsequent training of the machine learning solution difficult (Miceli et al., 2020). Additionally, it's crucial to have a good way to integrate annotations from multiple annotators, carefully considering how to deal with labeling merging in unmatched results when and the seniority of the experts. Furthermore, as labeling is an expensive task, unsupervised or semi-supervised techniques could be employed to generate cheaper but potentially more consistent labels (dos Santos Ferreira et al., 2019).

To fully exploit the potential for personalized healthcare, collecting metadata may be necessary. However, current General Data Protection Regulation (GDPR) regulations impose limitations to ensure both data privacy and security. To address this challenge, several approaches have been proposed, including federated machine learning, multi-party computation, and differential privacy. These methods provide a win-win solution by enabling the collection of necessary data while preserving the privacy and security of sensitive information (Brauneck et al., 2023).

This can be achieved through training programs, workshops, and online resources that provide guidance on best practices for data acquisition, preprocessing, and analysis in neuroimaging (Borghi and Van Gulick, 2018). The development of established protocols in standardization and analysis, such as those proposed for other neurodegenerative diseases like the Alzheimer's Disease Neuroimaging Initiative (ADNI) database (Wyman et al., 2013), can also serve as important models for promoting consistency and reliability in neuroimaging research.

## Limitations associated with machine learning/deep learning

### Generalization issues that hinder transferability

Neural networks (NNs) have been shown to be highly effective in approximating complex functions and achieving accurate predictions by leveraging large and high-quality datasets. However, despite demonstrating good performance on the training data, there is no guarantee that the model will continue to perform well on new and unseen data. This phenomenon, known as overfitting, occurs when the model is too closely tailored to the training data, and thus, is not generalizable to new data. Out-of-distribution and out-of-domain examples can cause neural networks to learn incorrect correlations and make inaccurate predictions. Common causes of overfitting include domain shift (Kondrateva et al., 2021), task mismatch (Castro et al., 2020), and catastrophic forgetting (Gupta et al., 2021). Poor generalization can lead to unreliable and incorrect predictions on real-world tasks where the data distribution may differ significantly from the training data (Yagis et al., 2019; Ge et al., 2023). In the context of CAD for PD, this may result in incorrect predictions that could lead to misdiagnosis or failure to detect the disease, ultimately resulting in incorrect treatment or delayed diagnosis.

To reduce overfitting, techniques such as regularization (Kukačka et al., 2017) and early stopping (Prechelt, 1998) can be employed. Data augmentation techniques can also expand the dataset size and improve internal generalization (Chlap et al., 2021). However, data augmentation alone cannot address demographic representativeness issues. Thorough internal and external validation is essential to ensure reliable and accurate model performance, especially for new and unseen data (Garcia Santa Cruz et al., 2021). Cross-validation techniques such as stratified cross-validation (Zeng and Martinez, 2000) and leave-one-out cross-validation (Hastie et al., 2009) can be used for internal validation, while external validation can be achieved through external datasets. These techniques can enhance model transferability and promote generalizability.

Additionally, when dealing with a small sample size, as is often the case in biomedical datasets, splitting the dataset for cross-validation may lead to a loss of the algorithm's generalization capacity. This limitation arises from the fact that when the sample size is small, dividing it into training and validation sets further reduces the amount of data available for training, potentially hindering the algorithm's ability to generalize well. Despite the conventional wisdom that attributes this small generalization error to properties of the model family or regularization techniques used during training (Zhang et al., 2021), it has been demonstrated that even with explicit regularization, state-of-the-art convolutional networks can fit random labeling of the training data, suggesting that these models have enough capacity to memorize the training data. A potential solution is to employ distribution-free performance bounds (Jakubovitz et al., 2019), which have been successfully implemented in neuroimaging (Górriz et al., 2019; Jimenez-Mesa et al., 2023).

To address data drift, various techniques can be employed. Calibration techniques (Wald et al., 2021) and appropriate metrics for evaluating model generalization (Jiang et al., 2019) can be used. Additionally, selecting the appropriate model architecture and hyperparameters can significantly enhance the model's generalization ability. Techniques such as grid search or Bayesian optimization (Kandasamy et al., 2018) can be employed to optimize hyperparameters. Furthermore, transfer learning has been demonstrated as an effective approach for improving model generalization, particularly when working with limited data (Yosinski et al., 2014).

Another big issue that can hinder the generalization of models is when they fail to learn the desirable patterns that characterize the phenomena we are trying to model, and instead learn spurious correlations. This can result in the model learning potential confounders, colliders, and other unwanted biases.

To address these issues, it is important to carefully evaluate the data used to train the model, identify potential confounders and colliders biases, and use appropriate statistical methods to account for them (Wang et al., 2018). Additionally, confounding removal strategies such as domain adaptation techniques can be employed during the harmonization phase (Dinsdale et al., 2021) and during the training process (Qin et al., 2020). Finally, it is crucial to regularly monitor the performance of the model and validate its results against independent and temporally updated data sets to identify and correct potential unwanted biases (Tamburri, 2020).

#### Algorithmic bias

This can be considered an extension of a generalization issue. Algorithmic bias is another significant challenge in ML, particularly in medical diagnosis and other decision-making applications. Societal biases and data acquisition biases can result in systematic and repeatable errors that lead to unfair outcomes and lower accuracy for certain groups (Ricci Lara et al., 2022). It is essential to address these biases in the design, training, and evaluation of NNs to ensure fairness and avoid perpetuating existing inequalities. These biases can result in systematic and repeatable errors, leading to unfair outcomes that favor certain groups over others, ultimately lowering the accuracy of the recommendation for some patient groups, particularly when there are racial biases. These biases can originate from existing inequality (Ricci Lara et al., 2022) or can also stem from selection bias introduced during the acquisition process (Garcia Santa Cruz et al., 2022b).

For example, Obermeyer et al. (2019) identified some systemic conditional disparities in risk scores based on the medical history of Black patients. In such cases, bias-correcting techniques can be employed (Wiens et al., 2020). Bias can also be introduced during the data acquisition process, resulting in technical debt and downstream effects known as data cascades (Sambasivan et al., 2021). Moreover, it is essential to address the issue of unwanted biases in the data used for current AI systems, as these systems not only have the risk of making incorrect predictions, but also of perpetuating and amplifying biases present in the data (Zhao et al., 2017).

The ML community has made interdisciplinary efforts to address the aforementioned issues, leading to the development of a range of solutions that fall under the umbrella of fairness (Mehrabi et al., 2021). By implementing such strategies in algorithm design, training, and evaluation, performance across groups can be improved, thereby mitigating the risk of unfairness in the final solution. These solutions typically target characteristics that have traditionally been the source of unfair discrepancies, such as gender and ethnicity. However, it is also crucial to ensure that algorithms perform well in cases where diseases have subgroups, such PD subtypes (Thenganatt and Jankovic, 2014) and varying degrees of disease penetrance (Espay et al., 2017). In such cases, similar metrics can be used, with the subgroups or disease penetrance considered as protected attributes.

### Need for better interpretability

Another significant issue with NNs is their inability to accurately represent uncertainty in their predictions (Abdar et al., 2021). Since NNs are deterministic, they cannot capture the notion of what they know and what they do not know, or the confidence level of their predictions. Furthermore, current NNs are limited to accessing the knowledge contained in the dataset. This lack of uncertainty estimation can lead to overconfidence in their predictions, which can be problematic in critical applications such as medical diagnosis or self-driving cars.

Before implementing CAD systems for PD as decision-making tools in clinical practice, it is essential to establish an interpretability strategy (Chan et al., 2020). CAD systems with low interpretability can have severe consequences, such as decreased trust and acceptance among clinicians and patients, misdiagnoses, and ineffective treatment strategies. A transparent and understandable model can help clinicians validate the system's predictions and ensure that the model is not making decisions based on spurious correlations or biases. Additionally, interpretability can help researchers gain new insights into PD and refine the diagnostic criteria.

The lack of uncertainty estimation can lead to overconfidence in their predictions, which can have severe consequences such as misdiagnoses and ineffective treatment strategies. Therefore, it is essential to establish an interpretability strategy before implementing CAD systems in clinical practice. Furthermore, the limitations of current explainability methods used in ML decision-making systems suggest that unless there are significant advances in explainable ML, we must treat these systems as black boxes, justified by their reliable and experimentally confirmed performance. Finally, it is recommended that healthcare workers exercise caution when using explanations from ML systems and regulators be judicious in listing explanations among the requirements needed for clinical deployment of ML (Ghassemi et al., 2021).

Recent regulations, such as the GDPR in the European Union (EU), emphasize the right to be informed and the right to contest an automated decision. In such cases, interpretability of AI becomes crucial for auditing the decision-making of automated agents such as ML models. In particular, Article 22 of the GDPR deals with the rights related to automated individual decision-making since data subjects cannot be subject to a decision based solely on automated processing (Council of European Union, 2016). Additionally, Articles 12 and 13 specify the right to be informed about the use of their data in an easily understandable and accessible manner. The most common use cases for participant data fall into two main scenarios: (1) data subjects provide their data to train AI models, and (2) data subjects receive a result from an AI model after providing some data. The first scenario requires informing the participants about the purpose and usage of their data. However, the second scenario requires additional clarification, as the participants should understand how a decision was made and, in particular, which input data was relevant for obtaining a specific result.

To meet the above requirements, ML solutions must be designed with transparency in mind. Some ML approaches produce models that are inherently easier to inspect. Decision tree predictive models are popular due to their intelligibility and simplicity. However, this approach does not suit all tasks. Essentially, models optimize a function that draws the boundary to separate the given classes (e.g., healthy vs. diseased) by grouping nearby instances. However, the definition of proximity differs across ML learners and interpretability measures become complex. For instance, random forest methods constitute an evolution of decision trees but at the cost of intrinsic interpretability since their internal model consists of a collection of decision trees, obfuscating the “reasoning” of the trained model (Nair et al., 2013). Another approach includes tracking the decision-making process on CNNs. For instance, Magesh et al. (2020) employ Local Interpretable Model-Agnostic Explainer (LIME) to increase the explainability of CNN-based models for PD diagnosis. Two key elements to improve interpretability are solutions to improve model *explainability* and model *uncertainty*.

#### Model explainability

Model explainability refers to the ability to understand how a ML model makes its predictions. It is important because in critical applications, such as healthcare or finance, it is necessary to understand why the model makes certain decisions, especially when human lives or significant resources are at stake. For example, if a model is predicting whether a patient has PD or make a recomendation about the treatment, it is important to know which factors the model is considering in its decision-making process.

Explainability and interpretability terms are is frequently used interchangeably and for this work, we do not distinguish between them. Of course, interpretability tools vary across ML methods, but there are some important methods worth mentioning that can facilitate the interpretability of the results. Molnar (2020) provides an overview of the available techniques for ML interpretability. The author distinguishes between intrinsic and *post hoc* methods. The first group concerns models whose simple structure permits human interpretation, e.g., short decision trees. The second group of methods are used after model training. Additionally, the author divides interpretability methods into model-specific and model-agnostic. The author provides yet another criterion to separate the methods into two groups, i.e., local (for methods that explain a particular result) and global (for methods that explain the whole model behavior) interpretability.

Aside from the above, solution design can impact model interpretability as well. Often models are designed in an end-to-end way that attempts to map input data with the final result with a single model. For instance, a medical imaging CAD system can be designed as a chain of several models, with the first dedicated to finding pathologies and the subsequent models mapping pathologies to diseases or conditions (e.g., through several one class classifiers) (Vega, 2021). This approach eases solution maintainance and increases interpretability, allowing inspection of the intermediate results.

To address this challenge, researchers have proposed various methods for interpreting and explaining the decisions of ML models, including model-agnostic techniques such as LIME (Visani et al., 2022) and SHapley Additive exPlanations (SHAP) (Kaur et al., 2020), as well as model-specific approaches such as attention mechanisms (Vaswani et al., 2017) and gradient-based attribution methods (Ancona et al., 2019).

#### Model uncertainty

In the context of medical diagnosis, the concept of model uncertainty plays a crucial role in determining the degree of confidence or uncertainty that a model has in its predictions. This consideration is particularly pertinent given the high stakes involved in clinical decision-making. The degree of certainty or uncertainty in a model's output is a crucial factor in determining appropriate actions to be taken based on the model's predictions. As such, accounting for model uncertainty can enhance the transparency and reliability of medical diagnosis, leading to more effective treatment strategies and improved patient outcomes.

Uncertainty in ML can stem from multiple sources. Some of them include data variance, lack of representativity in the data sample, label noise, and the intrinsic imperfections of any ML model developed from such data. The literature also refers to these types of uncertainty as systematic, aleatoric and epistemic, (Hüllermeier and Waegeman, 2021; Gal et al., 2022). Most of these issues cannot be fixed *a posteriori* and must be avoided through careful data acquisition design. However, documenting uncertainty sources and quantifying its magnitude in data, labels and model is of uttermost importance, in the same way we should document other aspects such as the representativity of the sample. This information is key to assess the generalization power of the solutions to new settings. For instance, reporting probability estimates together with the model prediction can indicate the model prediction confidence. However, these estimates may not accurately reflect model uncertainity calling for calibration methods (Lemay et al., 2022).

### Costly systems to develop and maintain

ML solutions are also expensive in terms of data and computation. Developing and training ML models requires a substantial amount of data, computing power, and specialized expertise. Acquiring large and diverse datasets can be challenging, and data collection, cleaning, and preprocessing can be time-consuming and labor-intensive (Ngiam and Khor, 2019). Moreover, the development and training of ML models often require specialized hardware, such as Graphics Processing Units (GPUs), which can increase energy consumption and carbon footprint (Patterson et al., 2021). It is important to consider the environmental impact of ML and take steps to reduce it, such as using energy-efficient hardware or exploring alternative training methods that require fewer computing resources (Wang et al., 2020).

In addition, ML models require ongoing monitoring, updating, and maintenance to ensure their continued accuracy. As data changes over time, the models may need to be retrained or updated to account for new patterns or trends. In the case of PD, this can be particularly challenging due to the variability in disease progression across patients, making it difficult to develop models that accurately capture the underlying patterns of the disease. Furthermore, implementing ML systems in clinical practice requires careful consideration of regulatory and ethical concerns to ensure patient safety and privacy. ML models used in clinical practice must undergo rigorous testing and validation to ensure their safety, efficacy, and reliability. The validation process involves evaluating the model's performance on independent datasets and comparing it to other established diagnostic methods (Liu et al., 2019). Additionally, models must be regularly audited to identify and mitigate biases and errors that may affect their performance (Reddy et al., 2020).

To address the challenges of cost and development associated with ML, there has been a concerted effort to develop open-source platforms and tools that make ML more accessible to researchers and clinicians. For instance, several open-source libraries, including TensorFlow (Abadi et al., 2016a), PyTorch (Paszke et al., 2019) and MONAI (Cardoso et al., 2022) provide pre-built ML models and algorithms that can be readily adapted and customized for specific applications. In addition, cloud computing platforms, such as Amazon Web Services and Google Cloud, offer scalable and cost-effective solutions for training and deploying ML models. Moreover, there is a growing trend toward collaborative and decentralized approaches to ML development (Castiglioni et al., 2021). One such approach is federated learning, which allows multiple parties to train a shared ML model without sharing their data, thus preserving data privacy and security (Tedeschini et al., 2022). Another approach is to use blockchain technology to create decentralized ML models that are transparent, auditable, and resistant to tampering (Neelakandan et al., 2022). These developments are expected to enhance the accessibility and affordability of ML solutions, thereby facilitating their wider adoption and implementation in clinical practice.

### Security and privacy challenges

Healthcare institutions are frequent targets of malicious hackers, resulting in data breaches and ransomware attacks (Branch et al., 2019; Devi, 2023). In March 2023, the Hospital Clinic de Barcelona, which serves half a million people, suffered a ransomware attack by the RansomHouse group, resulting in the theft of 4.4 TB of data (Toulas, 2023). Healthcare ML models often deal with very sensitive patient data, making them attractive targets for malicious attacks.

Adversarial training is a technique used to improve the robustness of ML models against adversarial attacks (Madry et al., 2017). It involves training the model on adversarial examples generated by an adversary system to make the model more resilient to similar attacks. However, these techniques can also be used maliciously. Adversarial attacks can cause the model to make incorrect predictions, which could potentially expose personal information from healthcare ML models. In membership inference attacks, an adversary attempts to determine whether a particular individual's data was used to train a machine learning model (Hu et al., 2022). In model inversion attacks, the aim is to reconstruct an individual's data from the outputs of a machine learning model. This can be achieved by generating adversarial examples that maximize the likelihood of the individual's data, given the model outputs (Fredrikson et al., 2015). These attacks highlight the need for robust security measures to be in place to protect healthcare ML models from malicious attacks.

The most effective safety measure for healthcare ML models is to restrict access to the trained models to authorized personnel. Additionally, privacy-preserving machine learning techniques such as differential privacy and homomorphic encryption can help prevent these attacks (Abadi et al., 2016b; Aono et al., 2017). It is advisable to take a proactive approach to healthcare privacy and security during the solution design instead of a reactive approach (Song et al., 2019; Bhuyan et al., 2020).

## Concluding remarks and perspectives

During recent years, both the ML and the medical community have begun to consider data quality as the most crucial factor impacting the performance of the solutions and their robustness, (Sambasivan et al., 2021). However, acquiring high-quality data, building a suitable model for the task, and determining the appropriate use for such models, remain challenging objectives toward clinically relevant models. In particular, Sambasivan et al. (2021) insist on building incentive structures across all stakeholders, stating that “many practitioners described data work as time-consuming, invisible to track, and often done under pressures to move fast due to margins–investment, constraints, and deadlines often came in the way of focusing on improving data quality.” Data bootstrapping is yet another source of issues in high-stakes AI domains, as many researchers begin the AI/ML work employing existing data or data collected for non-AI purposes that leads to poor generalization. It is essential to ensure that ML models are rigorously validated and tested before they can be used in clinical practice. The employment of datasets from multiple independent studies can boost the statistical power and lead to more accurate, reliable and reproducible research. In ML, a common practice to this end is to mix several datasets. However, if the mixed datasets do not share certain degree of methodological similarity, biases may be introduced due to differences in acquisition, preprocessing or annotation.

The circumstances previously described hinder the availability of large datasets containing multiple imaging modalities as large datasets often consist of multi-center cohorts employing different acquisition devices, protocols and pipelines. Overall, developing and maintaining ML systems for clinical practice can be a costly and time-consuming process that requires significant expertise and resources. However, the potential benefits, such as improved diagnosis and treatment outcomes for patients with PD, make it a worthwhile investment. The use of CAD tools to interpreted brain images is the context of PD is very promising. However, as previously mentioned, these solutions will be used as assisting tools in a very specific context and under specialized supervision and must pass a series of verification before they can be used, as is the case with other medical products or treatments. To achieve this, the models must be accompanied by interpretability methods to ensure that clinicians can understand how the model makes its predictions.

While this review focuses primarily on brain imaging, it has become increasingly clear that a single measure is unlikely to be sufficient for diagnosing PD in the foreseeable future. Instead, a combination of measures will likely be necessary. The most critical aspect of a biomarker is not its ability to diagnose PD in its early stages, but rather its ability to reflect the disease's pathogenesis and progression. By using a multimodal approach that combines various imaging biomarkers, clinicians can make early, accurate, and objective diagnostic decisions, identify neuroanatomical and pathophysiological mechanisms, and evaluate disease progression and therapeutic responses to drugs in clinical trials.

A common approach in developing multimodal CAD systems involves combining multiple imaging modalities as well as leveraging ensemble learning to integrate data from various sources for obtaining the final result. A concrete example of a multimodal approach in PD is the employment of multiple modalities to characterize a specific pathological process in certain regions of the brain. For instance, multimodal approaches employing hybrid images created through the integration of different MRI parameters offer a valuable tool. By combining T1-, T2*-, and diffusion-weighted MRI, Barbagallo et al. (2016) proposed to enable the detection and analysis of macro- and micro-structural abnormalities in the nigrostriatal pathway. The key benefit of integrating hybrid images enhances the accuracy and reliability of CAD systems by capturing diverse aspects of neurodegeneration.

Another example of a multimodal approach consists in combining MRI techniques, particularly those visualizing pathological changes in the substantia nigra using diffusion, iron-sensitive susceptibility, and neuromelanin-sensitive sequences, which offer a more accessible imaging tool. However, these techniques may be insufficient for phenotyping or prognostication due to the heterogeneous nature of PD resulting from extranigral pathologies. In Siderowf et al. (2023) highlight the emerging role of retinal optical coherence tomography as a non-invasive technique to visualize structural changes in the retina, which can serve as potential biomarkers for early diagnosis and prognostication in PD. Ensemble learning, a popular technique employed in multimodal CAD systems, plays a crucial role in fusing information from diverse data sources. Through ensemble learning, multiple models are trained independently on different subsets of data or using distinct feature representations. Ensemble learning had been successfully applied in PD classification using multimodal voice and speech data (Ali et al., 2021).

Recent promising markers that use the biochemistry of alpha-synuclein seed amplification assays have shown potential (Siderowf et al., 2023). For instance when recommending DBS as a therapy option for PD, it is important to consider genetic information, specifically whether the patient is a carrier of mutations in the glucocerebrosidase (GBA) gene. PD patients with GBA mutations are at particularly high risk for cognitive impairment with DBS due to dysfunction of the glucocerebrosidase (GCase) enzyme, resulting in more rapid accumulation and spread of Lewy bodies. Recent research has shown that PD patients experience cognitive impairment after DBS, and this risk is even greater for those with GBA mutations. Therefore, models that assist with therapy recommendations for PD patients should carefully evaluate whether patients are carriers of GBA mutations before recommending DBS as a treatment option (Pal et al., 2022).

Furthermore, there is an extended literature of ML models that have the potential to become CAD systems in the future from diagnosis and monitoring of PD, by providing more accurate and objective measurements of motor symptoms and disease progression. However, until this model are properly validated there are far to be ready for its used in clinical settings to ensure their safety and effectiveness in clinical practice.

Ultimately, our review emphasizes the critical importance of taking a multidisciplinary approach and putting in extensive effort during the data preparation and clinical validation phases of developing ML models. It is crucial to recognize that proper design and clinical validation may be undervalued in comparison to the training of ML models, but they are indispensable for data-driven CAD solutions that are safe for a clinical use. We hope that this review will inspire both future users and developers of these systems in the context of MRI for PD.

## Author contributions

BG conceived, structured, and wrote the manuscript. AH and FH supervised the manuscript. All authors contributed to the article and approved the submitted version.
